# Long‐term results of elective mucosal irradiation for head and neck cancer of unknown primary in Chinese population: The EMICUP study

**DOI:** 10.1002/cam4.2856

**Published:** 2020-01-17

**Authors:** Shengjin Dou, Rongrong Li, Lin Zhang, Zhuoying Wang, Li Xie, Chenping Zhang, Guopei Zhu

**Affiliations:** ^1^ Radiotherapy Division Department of Oral and Maxillofacial‐Head Neck Oncology Shanghai Ninth People’s Hospital College of Stomatology Shanghai Jiao Tong University School of Medicine Shanghai China; ^2^ National Clinical Research Center for Oral Diseases Shanghai China; ^3^ Shanghai Key Laboratory of Stomatology Shanghai Research Institute of Stomatology Shanghai China; ^4^ Department of Head and Neck Surgery Fudan University Shanghai Cancer Center Shanghai China; ^5^ Clinical Research Center Shanghai Jiao Tong University School of Medicine Shanghai China; ^6^ Department of Oral and Maxillofacial‐Head Neck Oncology Shanghai Ninth People’s Hospital College of Stomatology Shanghai Jiao Tong University School of Medicine Shanghai China

**Keywords:** head and neck, intensity‐modulated radiation therapy, lymph node metastasis, mucosal irradiation, radiotherapy, unknown primary

## Abstract

**Objective:**

Controversy still exists regarding the volume of radiation for head and neck cancer of unknown primary (HNCUP). Theoretically, elective mucosal irradiation (EMI) should achieve a balance between survival and toxicity. This prospective study was conducted to evaluate the long‐term benefit of EMI in Chinese HNCUP patients.

**Methods:**

A phase II, single‐arm trial was performed at two centers in China. HNCUP patients with pathologically confirmed metastatic squamous cell carcinoma or poorly differentiated carcinoma were enrolled. Patients with metastatic lymph nodes limited to level IV and/or the supraclavicular fossa were excluded. The EMI approach was specifically customized to Chinese patients by differentiating HNCUP as putative nasopharyngeal carcinoma (NPC) or non‐putative NPC. The primary endpoint was 3‐year mucosal recurrence‐free survival (MRFS).

**Results:**

A total of 48 patients were enrolled between 02/02/2010 and 08/01/2018; 46 patients were analyzed, including 24 putative NPC and 22 non‐putative NPC patients. No primary recurrence was observed during a median follow‐up period of 70 months, and only 1 patient experienced out of field recurrence in the contralateral neck. The 3‐year MRFS was 90.6% (95%CI: 76.4%‐96.4%). The 5‐year MRFS, regional‐recurrence free survival (RRFS) and overall survival (OS) were 90.6% (95%CI: 76.4%‐96.4%), 86.0% (95%CI: 71.1%‐93.7%), and 90.6% (95%CI: 76.4%‐96.4%), respectively. No grade 4 acute or late toxicities occurred, and the most frequent grade 3 acute toxicity was oral mucositis (45.7%).

**Conclusion:**

To the best of our knowledge, this is the first prospective study to evaluate the long‐term outcomes of EMI in Chinese HNCUP patients. Excellent MRFS and OS rates were observed. Further randomized studies are warranted.

## Graphical abstract

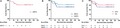

## 1 INTRODUCTION

Head and neck cancer of unknown primary (HNCUP) represents a heterogeneous group of patients with metastatic cancer in cervical lymph nodes (LNs) and clinically undetectable primary tumor sites, accounting for approximately 1%‐5% of all head and neck cancers.^1^, ^2^, ^3^ As HNCUP constitutes a favorable‐risk cancer of unknown primary (CUP) in patients,^2^, ^3^ aggressive and customized treatment should be provided to assure the greatest chance for cure while minimizing toxicity. Due to a lack of definite evidence from robust prospective trials, treatment recommendations for HNCUP are not specific, and no specific guidelines exist regarding target volumes. Although comprehensive radiotherapy (RT) of both sides of the neck and the entire pharyngeal mucosa has been commonly adopted,^4^, ^5^, ^6^, ^7^ controversy remains regarding whether the treatment volumes should include the mucosal area in addition to the neck node levels, and what extent of mucosa area should be irradiated: total mucosal irradiation (TMI, including nasopharynx, oropharynx, larynx, and hypopharynx) or elective mucosal irradiation (EMI).^5^, ^8^, ^9^ Although TMI aims to eradicate the potential cancer that may not be discovered during the diagnostic workup,^5^, ^7^, ^10^, ^11^, ^12^, ^13^ toxicity, and long‐term morbidity (mostly dysphagia and xerostomia) caused by extensive irradiation volumes should not be ignored even in the intensity‐modulated radiation therapy (IMRT) era.

The current trend in the management of HNCUP is EMI, in which the radiation field is limited to the mucosal area that is most likely the primary site. A high incidence of nasopharyngeal carcinoma (NPC) occurs in China. Therefore, in our practice, HNCUP is classified as putative NPC and non‐putative NPC based on clinical findings. Putative NPC is treated by elective irradiation to the nasopharynx and bilateral neck. Non‐putative NPC is treated by upfront neck dissection (ND) followed by irradiation to the unilateral oropharynx, hypopharynx, and supraglottic structures and neck LN levels; sparing the nasopharynx, oral cavity, vocal cords, subglottic larynx, and cervical esophagus.^14^ Using this elective treatment approach, we anticipate encouraging mucosal control with acceptable toxicities, especially in late toxicities. Thus, a phase II EMICUP study was conducted to evaluate the long‐term benefit of EMI in HNCUP patients.

## 2 MATERIAL AND METHODS

### 2.1 Patients

The eligibility criteria for this multi‐center phase II study were as follows: (a) pathologically confirmed metastatic squamous cell carcinoma or poorly differentiated carcinoma of the neck LNs; (b) patients underwent a comprehensive workup, including a complete head and neck examination, magnetic resonance imaging (MRI), and/or computed tomography (CT) of the head and neck region, and panendoscopy with directed biopsies with no primary site identified; (c) stage N1‐N2c disease (7th AJCC/UICC staging system); (d) age of 18‐70 years; (e) ECOG performance status 0‐1; (f) no prior treatment; (g) no distant metastasis and (h) adequate hepatic, renal, and hematological function. Patients with metastatic LNs limited to level IV and/or the supraclavicular fossa were excluded. The trial was approved by the institutional review board. Written informed consent was obtained from all trial participants before enrollment.

### 2.2 Study design

**Figure 1:**
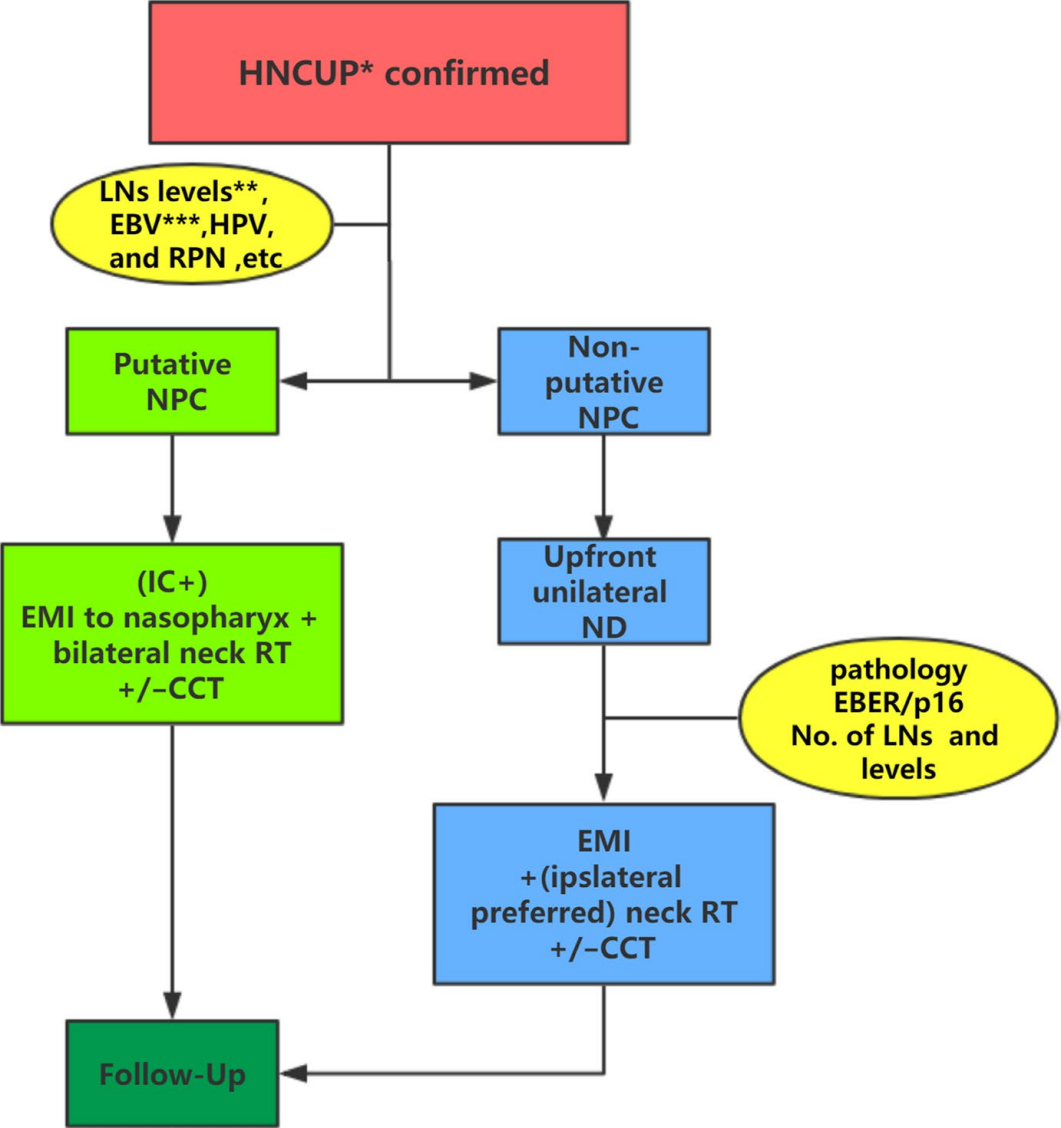
Treatment principle of the how to deliver EMI approach for putative NPC and non‐putative NPC patients. Notes: *pathologically confirmed squamous cell carcinoma and poorly differentiated epithelial tumors; **radiology evaluation with enhanced MRI or CT; ***EBV VCA‐IgA or EBV DNA (preferred). Abbreviations: CCT, concurrent chemoradiotherapy; EBER, EBV‐encoded RNA; EBV, Epstein‐Barr virus; EMI, elective mucosal irradiation; HNCUP, head and neck cancer of unknown primary; HPV, human papillomavirus; IC, induction chemotherapy; LN, lymph node; ND, neck dissection; NPC, nasopharyngeal carcinoma; RPN, retropharyngeal node; RT, radiotherapy

This was a multi‐center, single‐arm, phase II trial performed in accordance with the Declaration of Helsinki and good clinical practice guidelines. The treatment for all enrolled patients was decided by a multidisciplinary team including radiation oncologists, medical oncologists, surgeons, pathologists, and radiologists. The treatment principles are shown in Figure 1. Putative NPC was presumed if the involved LN(s) was at level II (especially level IIb) and a retropharyngeal node (RPN) was involved or Epstein‐Barr virus (EBV) VCA‐IgA status was positive, but a nasopharynx biopsy failed to confirm NPC. Putative NPC patients were treated with concurrent chemoradiotherapy (CCT) with/without induction chemotherapy. Other patients were classified as non‐putative NPC and treated by upfront ND followed by RT with/without concurrent chemotherapy. Platinum‐based chemotherapy was recommended for induction chemotherapy and concurrent chemotherapy.

### 2.3 Pretreatment evaluations

Pretreatment evaluations included the following: a detailed medical history, an evaluation of performance status, and careful physical examination. Endoscopy of the nasopharynx, oropharynx, hypopharynx, and larynx under anesthesia was performed. Contrast‐enhanced MRI or CT scans of the head and neck, chest CT, abdominal ultrasound, and hematologic profile analysis were performed within 3 weeks of study entry. Positron emission tomography (PET) scans, CT scans of the abdomen, and bone scans were performed as clinically indicated. Tests for EBV VCA‐IgA were done for suspicious putative NPC patients without RPN involvement. At that time, we were not fully aware of the implications of disease associated with human papillomavirus (HPV) in non‐putative NPC patients. HPV or p16 tests in postoperative non‐putative NPC patients to determine HPV status were strongly recommended since 2013. Tests for EBV‐encoded RNA (EBER) were recommended since then in these patients to further confirm the diagnosis.

### 2.4 Radiation volumes and dose prescription

**Figure 2:**
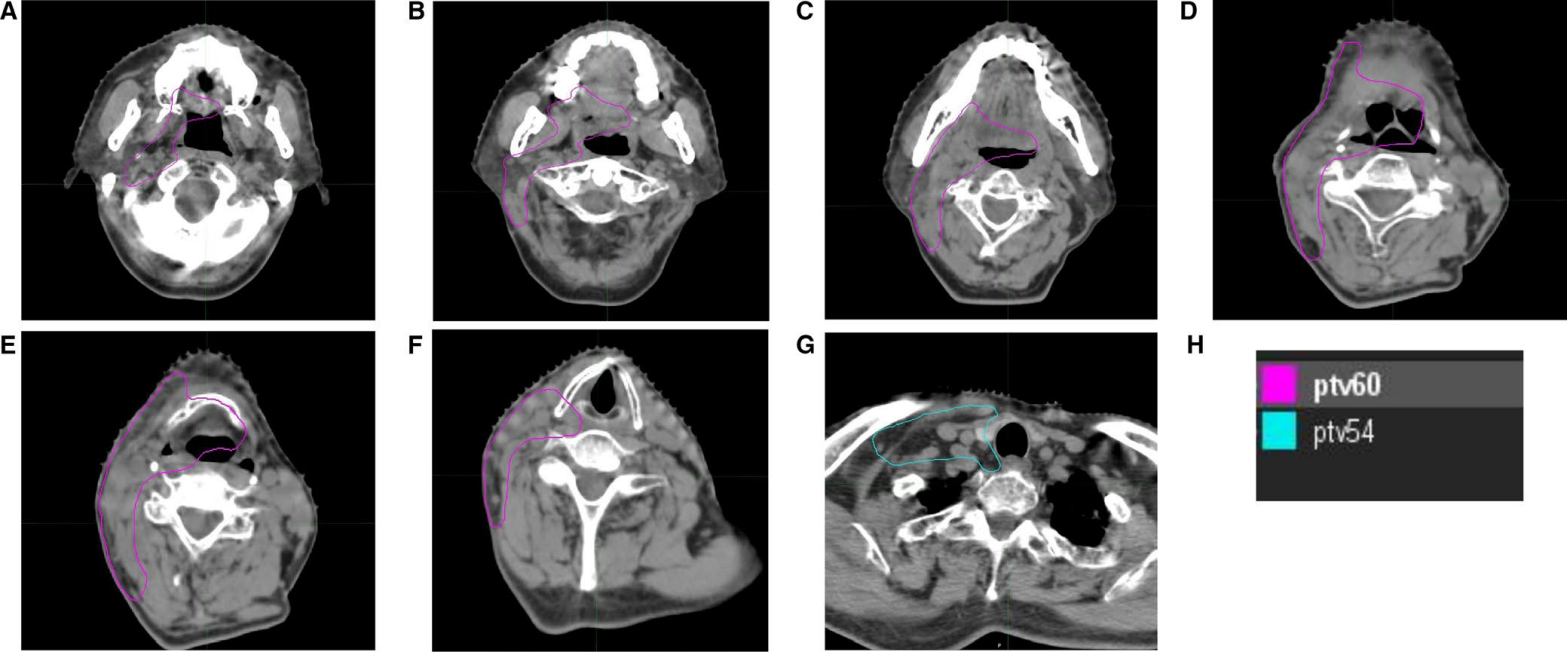
Target volume delineation standard for non‐NPC patients (planning target volume for elective mucosal irradiation). The target volume including the unilateral half of the oropharynx, the hypopharynx, the supraglottic structures and neck LN levels irradiation (unilateral neck irradiation was performed for N1, N2a disease <4 cm or ≥4 cm without extranodal extension (ENE), or N2b disease ＜3 positive LNs and within one LN level; bilateral neck irradiation was performed for N2c disease, N2a disease ≥4 cm with ENE, N2b disease with ≥3 positive LNs, or N2b disease with multiple levels of LNs), and the nasopharynx, oral cavity, vocal cords and cervical esophagus were excluded. Doses of 60 and 54 Gy in 30 fractions were administered to the putative mucosal region and high‐risk involved neck region and to the low‐risk neck region, respectively

All patients underwent CT‐based IMRT planning and EMI. For putative NPC patients, the radiation volume included the nasopharynx and bilateral cervical LNs. A dose of 66, 60, and 54 Gy in 30 fractions was given to gross tumor, the nasopharynx region and high‐risk bilateral involved neck, and the bilateral low neck, respectively. Residual LNs observed on follow‐up MRI at the end of treatment were treated with a local boost of 4‐6 Gy in 2‐3 fractions using photon or an electron beam. For non‐putative NPC patients, the target volume included the unilateral half of the oropharynx, the hypopharynx, and the supraglottic structures, and unilateral neck irradiation was performed for N1, N2a disease <4 cm or ≥4 cm without extranodal extension (ENE), or N2b disease ＜3 positive LNs and within one LN level; bilateral neck irradiation was performed for N2c disease, N2a disease ≥4 cm with extranodal extension, N2b disease with ≥3 positive LNs, or N2b disease with multiple levels of LNs. The nasopharynx, oral cavity, glottic larynx and cervical esophagus were spared. A dose of 60 and 54 Gy in 30 fractions was delivered to the putative mucosal region and the high‐ and low‐risk involved neck, respectively. (Figure 2).

### 2.5 Follow‐up

Patients were followed up every 3 months for the first 2 years after the completion of RT and every 6 months for the third to fifth years. A physical examination and ultrasound of the neck were performed at every follow‐up visit. Nasopharyngoscopy was performed at every follow‐up visit for putative NPC patients. MRI or CT of the head and neck, Chest CT and ultrasound of the abdomen were performed every 6 months. A bone scan was performed if clinically indicated.

### 2.6 Endpoints and statistical analysis

The primary study endpoint was 3‐year mucosal recurrence‐free survival (MRFS). MRFS was measured from the day after RT completion to the day of discovery of a mucosal primary lesion or death. We used a two‐sided log‐rank test to calculate the sample size. Because historical data showed a 3‐year MRFS rate of 64.3% in HUCUP patients treated by ND ± neck radiation without radiation to the mucosa,^14^ we assumed a 3‐year MRFS rate of 90% in this study. To detect such a difference, a minimum of 43 patients (type I error 5% and power 90%) was required. To allow for a 10% ineligible/loss rate, the sample size was estimated to be 48 patients.

The secondary endpoints included the 5‐year MRFS, 3‐year and 5‐year overall survival (OS), regional recurrence‐free survival (RRFS), disease‐free survival (DFS), distant metastasis‐free survival (DMFS), and acute and late toxicities. All time to event outcomes were measured from the day after RT completion to the date of the event. The Statistical Package for Social Sciences (SPSS) version 20.0 (SPSS Inc, Chicago, IL) was used to calculate the MRFS, OS, RRFS, and DMFS using the Kaplan‐Meier method. The Common Terminology Criteria for Adverse Events (CTCAE) v4.0 were used to evaluate the toxicities.

## 3 RESULTS

### 3.1 Baseline characteristics

A total of 48 patients were enrolled between 02/02/2010 and 08/01/2018 in two centers in China. Two patients were excluded from the analysis because one refused RT and one refused EMI and received RT to the ipsilateral neck. Twenty‐four patients were diagnosed with putative NPC and received RT to the nasopharynx and bilateral neck; the remaining 22 patients did not meet the criteria for putative NPC, were diagnosed with non‐putative NPC and received upfront ND and EMI as mentioned above. Table 1 shows the baseline characteristics.

**Table 1:** Baseline patient characteristics

	All patients (N = 46)	Putative NPC (N = 24)	Non‐putative NPC (N = 22)
Age (year)			
Median	57.5	53	58.5
Range	24‐75	25‐74	24‐75
Gender			
Female	39 (84.8%)	19 (79.2%)	20 (90.9%)
Male	7 (15.2%)	5 (20.8%)	2 (9.1%)
Performance status			
0	31 (67.4%)	15 (62.5%)	16 (72.7%)
1	15 (32.6%)	9 (37.5%)	6 (27.3%)
Tobacco exposure			
Never smoker	26 (56.5%)	15 (62.5%)	11 (50.0%)
Former smoker	16 (34.8%)	7 (29.2%)	9 (40.9%)
Current smoker	4 (8.7%)	2 (8.3%)	2 (9.1%)
Tobacco‐smoking history			
<10 pack‐years	5 (25.0%)	1 (11.1%)	4 (36.4%)
>10 pack‐years	15 (75.0%)	8 (88.9%)	7 (63.7%)
N Stage			
N1	7 (15.2%)	3 (12.5%)	4 (18.2%)
N2a	10 (21.7%)	7 (29.2%)	3 (13.6%)
N2b	19 (41.3%)	7 (29.2%)	12 (54.5%)
N2c	10 (21.7%)	7 (29.2%)	3 (13.6%)
Lymph node levels			
I	5 (10.9%)	2 (8.3%)	3 (13.6%)
II	44 (95.7%)	22 (91.7%)	22 (100%)
III	16 (34.8%)	9 (37.5%)	7 (31.8%)
IV	11 (23.9%)	5 (20.8%)	6 (27.3%)
V	5 (10.9%)	1 (4.2%)	4 (18.2%)
Pathology			
SCC	32 (69.6%)	12 (50.0%)	20 (91.9%)
PDC	14 (30.4%)	12 (50.0%)	2 (9.1%)
EBV/HPV status			
VCA‐IgA Positive	15/24	14/17	1/7
EBER positive	0/7	—	0/7
HPV positive	5/7	—	5/7
p16 positive	2/6	—	2/6
RPN			
Positive	10 (21.7%)	10 (41.7%)	0 (0.0%)
Negative	36 (78.3%)	14 (58.3%)	22 (100.0%)
ENE			
Positive	20 (43.5%)	8 (33.3%)	12 (54.5%)
Negative	26 (56.5%)	16 (66.7%)	10 (45.5%)
PET‐CT			
Yes	23 (50.0%)	10 (41.7%)	13 (59.1%)
No	23 (50.0%)	14 (58.3%)	9 (40.9%)

Note

Abbreviations: EBV, Epstein‐Barr virus; ENE, extranodal extension; HPV, human papillomavirus; PDC, Poorly differentiated carcinoma; PET‐CT, positron emission tomography‐computed tomography; RPN, retropharyngeal node; SCC, squamous cell carcinoma.

For all 46 patients, the median age was 57.5 years (range: 25‐75 years). Nodal staging was distributed as follows: N1 15.2% (n = 7), N2a 21.7% (n = 10), N2b 41.3% (n = 19), and N2c 21.7% (n = 10). Level II LNs were involved in 95.7% (n = 44) of patients. ENE was diagnosed by imaging studies or pathological examination in 37 (48%) patients. PET‐CT was performed in 23 (50.0%) patients.

Among patients diagnosed with putative NPC, an EBV VCA‐IgA test was performed in 17 patients, and 14 patients had positive results. Other ten patients with negative or unknown EBV VCA‐IgA had RPN involvement, and 8 cases were pathologically confirmed.

Among non‐putative NPC patients, none of seven patients who underwent EBER detection had positive results. Nine patients underwent either HPV or p16 tests to determine HPV status. Three patients were positive for only the HPV test, and two patients were positive on both tests (Table 1).

### 3.2 Treatment compliance

The treatment of patients is summarized in Table 2. All patients completed the scheduled EMI with a median time of 45 days (44‐50 days). Among putative NPC patients, seven received neck LN boost for residual LNs at the end of treatment, 4 received 4 Gy in two fractions, and 3 received 6 Gy in 3 fractions.

**Table 2:** Treatment of two groups

	Putative NPC	Non‐putative NPC
	（N = 24）	（N = 22）
ND	—	22 (100%)
Ipsilateral		19 (86.4%)
Bilateral	—	3 (13.6%)
RT		
Nasphaynx + bilateral neck	24	—
EMI + bilateral neck	—	13 (59.1%)
EMI + unilateral neck	—	9 (40.9%)
RT dose		
66Gy/30Fx (no boost)	22 (91.7%)	1 (4.5%)^a^
60Gy/30F (no boost)	2 (8.3%)^b^	21 (95.5%)
Neck LN boost	7 (29.2%)	0 (100%)
Chemotherapy	24 (100%)	12 (54.5%)
Induction	15 (62.5%)	0 (0%)
Concurrent	10 (41.7%)	12 (54.5%)

Note

Abbreviations: EMI, elective mucosal irradiation; LN, lymph node; ND, neck dissection; RT, radiotherapy.

a 1 patient with one suspicious positive lymph node after ND before EMI.

b There was no gross disease after IC and there were no PTV of 66Gy in the neck in 2 patients.

Among non‐putative NPC patients, 9 received unilateral neck RT, and 13 received bilateral neck RT (3 with N2c disease, 8 with N2b disease, and 2 patients with N2a). In this group, 12 patients who presented with ENE received CCT, and all of these patients finished two cycles of platin‐based CCT.

### 3.3 Survival outcomes

**Figure 3:**
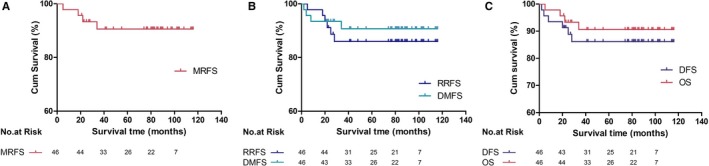
Kaplan‐Meier survivals of all HNCUP patients. (A) MRFC, RRFS and DMFS curves of all patients; (B): DFS and OS curves of all patients. Abbreviations: DFS, disease‐free survival; DMFS, distant metastasis‐free survival; MRFS, mucosal recurrence‐free survival; OS, overall survival; RRFS, regional recurrence‐free survival

The median follow‐up periods were 76 months (5‐116 months) for all patients and 76 months (21‐116 months) for all patients who remained alive. The 3‐year MRFS rate was 90.6% (95%CI: 76.4%‐96.4%). The 3‐year OS, RRFS, DMFS and DFS rates were 90.6% (95%CI: 76.4%‐96.4%), 86.0% (95%CI: 71.1%‐93.7%), 90.7% (95%CI: 76.4%‐96.4%), and 86.1% (95%CI: 71.1%‐93.7%), respectively. The 5‐year MRFS, OS, RRFS, DMFS and DFS rates were 90.6% (95%CI: 76.4%‐96.4%), 90.6% (95%CI: 76.4%‐96.4%), 86.0% (95%CI: 71.1%‐93.7%), 90.7% (95%CI: 76.4%‐96.4%), and 86.1% (95%CI: 71.1%‐93.7%), respectively. (Figure 3).

### 3.4 Failure patterns

No patient experienced primary emergence. Three patients experienced distant metastasis, all of whom died of the metastasis. Three patients developed isolated neck LN recurrence and 1 patient died of LN recurrence.

All 3 patients with distant metastasis were diagnosed with putative NPC and received radiation to the NPC and bilateral neck; 2 of these patients also received concurrent chemotherapy.

Of the 3 patients with neck recurrence, 1 was diagnosed with putative NPC and exhibited recurrence in the ipsilateral neck 20 months after RT; this patient died of neck recurrence. Two non‐putative NPC patients relapsed in the neck; one relapsed ipsilaterally 28 months after RT, and the other relapsed in the contralateral neck 25 months after RT. The latter received EMI and ipsilateral neck irradiation. These patients received salvage ND and remained disease free. Therefore, only 1 patient experienced out‐of‐field recurrence.

### 3.5 Toxicity

The acute and late toxicities are listed in Table 3. No grade 4 acute or late toxicity occurred. The most frequent grade 3 acute toxicity was oral mucositis (45.7%). The acute toxic effects of EMI were comparable to those in previous reports of CCT in NPC patients.^15^ The most frequent late toxic effect was xerostomia. Grade 3 xerostomia and dysphagia occurred in 2 (4.3%) and 4 patients (8.7%), respectively. No serious late effects such as osteonecrosis of the jaw or temporal lobe necrosis were observed.

**Table 3:** Treatment‐related toxicities

Toxicities	G1/2	G3	G4
Acute toxicities			
Oral mucositis	25 (54.3%)	21 (45.7%)	0 (0%)
Dermatitis	43 (93.5%)	3 (6.5%)	0 (0%)
Nausea/vomiting	22 (47.8%)	2 (4.3%)	0 (0%)
Liver dysfunction	4 (8.7%)	0 (0%)	0 (0%)
Kidney dysfunction	2 (4.3%)	0 (0%)	0 (0%)
Leukopenia	24 (52.2%)	1 (2.2%)	0 (0%)
Neutropenia fever	0 (0%)	0 (0%)	0 (0%)
Anemia	12 (26.1%)	0 (0%)	0 (0%)
Thrombocytopenia	8 (17.4%)	0 (0%)	0 (0%)
Late toxicities			
Xerostomia	15 (32.6%)	2 (4.3%)	0 (0%)
Hearing loss	3 (6.5%)	0 (0%)	0 (0%)
Cranial neuropathy	0 (0%)	0 (0%)	0 (0%)
Dysphagia	6 (13.0)	4 (8.7%)	0 (0%)
Osteonecrosis of the jaw	0 (0%)	0 (0%)	0 (0%)
Temporal lobe necrosis	0 (0%)	0 (0%)	0 (0%)

Note

Abbreviations: G, grade.

## 4 DISCUSSION

To the best of our knowledge, this is the first prospective clinical study focused on the clinical efficacy and toxicity of EMI in Chinese HNCUP patients. The encouraging results of this EMICUP study showed a 3‐year MRFS of 90.6%. Long‐term clinical benefit was demonstrated with a 5‐year MRFS rate of 90.6%, a 5‐year DFS rate of 86.1%, a 5‐year OS rate of 90.6%, and acceptable acute and late toxicities. The EMI approach was safe and feasible and seemed to balance tumor control and toxicity. In addition, this study also emphasized that putative NPC and non‐putative NPC should be defined and treated differently, as HNCUP includes a heterogeneous group of patients.

The relative rarity of HNCUP has prevented large‐cohort prospective studies; therefore, treatment recommendations are based on retrospective studies and treatment experience. In general, RT or surgery alone is recommended for N1 and N2a stage disease without ENE, and combined treatment consisting of surgery followed by adjuvant radiation (±chemotherapy) or primary chemoradiation (±post‐therapy ND) is required for more advanced disease.^2^ Although a single modality is recommended for N1 and N2a stage disease without ENE,^9^, ^16^ our retrospective analysis showed a mucosal failure rate of more than 15% after surgery alone, even in N1 patients,^17^ which suggests that RT with mucosal irradiation may also be beneficial for N1 and N2a patients.

A primary emergence rate of 25%‐30% with high rates of regional failure has been reported in patients treated with surgery alone^18^; however, whether the potential primary mucosal site should be electively treated is still controversial. A rational approach to EMI is to cover all mucosal regions that potentially harbor microscopic cancer while minimizing unnecessary radiation in normal tissue. In Asian countries, where NPC is a common malignancy, the nasopharynx should be carefully evaluated for the possibility of a primary site, especially for patients with disease limited to level II (IIb), level Va or with RPNs. RPNs are usually considered as the first‐echelon LNs for the nasopharynx. The rate of RPN metastases in NPC patients is reported as high as 94%.^19^ Du et al^20^ reported 49 HNCUP patients with RPNs treated with irradiation to the nasopharynx and bilateral cervical LNs, and only two patients experienced recurrence in the nasopharynx during a median follow‐up period of 37 months; no primary cancer other than the nasopharynx was detected. EBV DNA and EBER are also potential markers for putative NPC. Our data showed that no primary tumor emerged in 24 putative NPC patients, and only 1 patient experienced in‐field nodal recurrence, which demonstrated the excellent outcomes of EMI in putative NPC patients.

In non‐Asian countries, the most common sites harboring potential primary tumor in HNCUP patients are the base of the tongue and the tonsil,^21^ which may be related with HPV infection, as HPV‐related oropharyngeal cancer is often characterized as a bulky nodal disease with small primary lesions, and the incidence of HPV‐related HNCUP has also increased over recent years.^22^ Patients with HPV‐related HNCUP have similar OS and DFS to patients with T1‐2N1M0 HPV‐related oropharyngeal carcinoma.^23^ In non‐Asian countries, for HNCUP limited to level II (especially level IIA) or with a predominance of disease in level II and smaller LNs in level III, it is reasonable to treat patients with EMI to the oropharynx with nasopharynx and larynx omitted from treatment, particularly in patients with p16 + nodal disease. EMI to the oropharynx will likely play an important role in optimizing locoregional control and alleviating acute and late toxicity by sparing the aerodigestive tract.^24^, ^25^, ^26^

Regarding radiation dose, a dose of 60Gy were used in our study for the consideration that the high‐risk mucosal regions were hiding small cancers. IMRT techniques could spare patients from extensive toxicity.^7^ A moderate dose, such as 54 Gy, may also be sufficient for mucosal control,^5^ but 54Gy dose were delivered to the total mucosal regions for consideration of acute toxicities. This study showed the mucosal control rate of 100% with acceptable toxicities, indicating such approach is feasible.

Another disputed issue is whether ipsilateral neck RT or bilateral neck RT is necessary. Although a substantial consensus has been achieved for unilateral RT for N1 disease and bilateral RT for high‐risk disease, the choice of the extent of RT remains difficult and depends on the individual preferences of physicians. Although bilateral neck RT reduces neck LN recurrence, it also increases toxicity,^27^ and some studies failed to identify any survival benefit of this approach.^18^ In selected patients with N0‐N2b oropharyngeal cancer, ipsilateral elective radiation may result in equally excellent regional control,^28^ regardless of tumor HPV status.^29^ Although disputes still exist,^30^ upfront ND followed by radiation may result in improved local‐regional control in HNCUP patients,^31^ especially for Chinese patients with low incidence of HPV‐associated head and neck squamous cell carcinoma (HNSCC). Furthermore, the pathology was more accurate after surgery. Thus, we prefer upfront unilateral ND and ipsilateral neck RT with EMI in cases of unilaterally non‐bulky LNs (with bilateral neck irradiation for N2c disease, N2a disease ≥4 cm with ENE, N2b disease with ≥3 positive LNs, or N2b disease with multiple levels of LNs) in non‐putative patients.

The 8th edition TNM classification of HNCUP was recently changed and defines virus‐related tumors as T0 NPC/OPC. EBV and HPV/p16 status should be identified using histological methods. If evidence of EBV is present, T0 NPC staging should be applied. P16‐positive T0 oropharyngeal cancer staging should be applied if evidence of HPV and positive p16 overexpression are present. This change in staging for HNCUP provides a consensus that virus‐related HNCUP should be treated as NPC/OPC; therefore, EMI to the nasopharynx should be performed for EBV‐positive patients, and EMI to the oropharynx should be performed for HPV‐positive patients.

This study has some limitations. First, this study included patients treated between 2010 and 2018, and many changes in diagnostic approaches have occurred over this period, especially regarding the wide use of PET‐CT and narrow‐band imaging^3^, ^32^ in recent years. Second, the lack of HPV detection in some patients in the study is a limitation, as HPV‐associated oropharyngeal cancer has been recently recognized. However, the incidence of HPV infection in Chinese oropharyngeal cancer patients was reported to be only approximately 20%‐30%.^33^, ^34^

## 5 CONCLUSION

In conclusion, we report the long‐term outcomes of a prospective phase II study that evaluated EMI in Chinese HNCUP patients. Excellent rates of MRFS and OS were observed. Further prospective, randomized studies with more patients are warranted to investigate whether this EMI approach is beneficial.

## CONFLICT OF INTEREST

The authors declare that they have no competing interests.

## AUTHORS’ CONTRIBUTIONS

SD and RL contributed in the collection and analysis of data and drafting the manuscript. LZ and CZ helped with the initial design of the study. LZ and LX assisted with data collection and provided critical revision of the manuscript. GZ and ZW provided the conception of this study and the final approval of the version to be published. All authors have read and approved the final manuscript.

## Data Availability

The data that support the findings of this study are available from the corresponding author upon reasonable request.
